# Polypyrrole/polylactic acid nanofibrous scaffold cotransplanted with bone marrow stromal cells promotes the functional recovery of spinal cord injury in rats

**DOI:** 10.1111/cns.13135

**Published:** 2019-05-01

**Authors:** Raynald, Bing Shu, Xue‐Bin Liu, Jun‐Feng Zhou, Hua Huang, Jing‐Yun Wang, Xiao‐Dan Sun, Chuan Qin, Yi‐Hua An

**Affiliations:** ^1^ Institute of Laboratory Animal Science, Chinese Academy of Medical Sciences (CAMS), Comparative Medicine Centre Peking Union Medical College (PUMC) Beijing China; ^2^ Department of Functional Neurosurgery The Third Medical Centre, Chinese PLA (People's Liberation Army) General Hospital Beijing China; ^3^ Department of Neurosurgery, Beijing Sanbo Brain Hospital Capital Medical University Beijing China; ^4^ A State Key Laboratory of New Ceramics and Fine Processing, School of Materials Science and Engineering Tsinghua University Beijing China; ^5^ Key Laboratory of Advanced Materials of Ministry of Education of China, School of Materials Science and Engineering Tsinghua University Beijing China; ^6^ Beijing Neurosurgical Institute Capital Medical University Beijing China

**Keywords:** bone marrow stromal cell, functional recovery, PPy/PLA nanofibrous scaffold, spinal cord injury, transplantation

## Abstract

**Aims:**

The objective of this study was to analyze the efficacy of polypyrrole/polylactic acid (PPy/PLA) nanofibrous scaffold cotransplanted with bone marrow stromal cells (BMSCs) in promoting the functional recovery in a rat spinal cord injury (SCI).

**Methods:**

Female Sprague‐Dawley rats were randomly divided into three groups (n = 18/group): control group, PPy/PLA group, and PPy/PLA/BMSCs group. The SCI was induced in all rats. Consequently, rats in PPy/PLA/BMSCs group were transplanted with 1 × 10^5^ BMSCs after implantation of PPy/PLA, while those in the PPy/PLA group were implanted with PPy/PLA only; no implantation was performed in the control group. Six weeks after surgery, immunofluorescence microscopy, electron microscope, and polymerase chain reaction (PCR) techniques were performed to assess the changes in the injured spinal cord tissues.

**Results:**

Electrophysiology and locomotor function testing suggested that PPy/PLA nanofibrous scaffold cotransplanted with BMSCs could promote the functional recovery of the spinal cord. Six weeks after the operation, lower amount of scar tissue was found in the PPy/PLA group compared with the control group. Abundant neurofilament (NF) and neuron‐specific marker (NeuN) positive staining, and myelin formations were detected in the injured area. In addition, the transplantation of BMSCs not only improved the efficacy of PPy/PLA but also managed to survive well and was differentiated into neural and neuroglial cells.

**Conclusions:**

The implantation of PPy/PLA nanofibrous scaffold and BMSCs has a great potential to restore the electrical conduction and to promote functional recovery by inhibiting the scar tissue formation, promoting axon regeneration, and bridging the gap lesion.

## 1 INTRODUCTION

Spinal cord injury (SCI) is characterized by the loss of sensory and motor function caudal to the level of injury. Although many research studies have addressed the management of SCI, thus far no effective treatment has been developed. The main treatments for SCI include surgery, while the use of drugs and rehabilitation have shown to improve the neurological function to some extent. However, there are still many limitations for these treatment modalities. SCI causes a series of pathophysiological events, such as massive inflammation, edema, demyelination, cell death, vascular destruction, and glial scar, which affect the axons regeneration.^1^, ^2^ So far, various biomaterial scaffolds in the form of nerve guidance conduits have been widely developed and tested in vivo. These materials have the ability to improve functional recovery in nervous system injury by promoting new axon formation that span across the lesion gap.^3^, ^4^, ^5^, ^6^, ^7^, ^8^ Yet, the nerves conduction velocity (NCV) of regenerated nerves has shown to be significantly lower compared with the healthy nerves. Recent studies on biomaterials engineering have focused on obtaining the optimal functional recovery, and thus on examining scaffold materials that possess the ability to conduct electricity, and in turn promote nerve regeneration.^9^, ^10^ As a result, electro conducting polymers and their effects in promoting nerve regeneration have been widely investigated.

Polypyrrole (PPy) is a well‐known conducting polymer used in biomedical applications to enhance the nerve regeneration by electrical stimulation.^11^ PPy can easily be synthetized and offer good cytocompatibility and conductivity.^12^, ^13^, ^14^ In vitro studies have suggested that PPy can be used as a promising scaffold material for cell growth. For example, Schmidt et al^15^ have observed the promotion of neurite outgrowth from the cells after stimulating PC12 cells with PPy. Furthermore, Forciniti et al^16^ have observed Schwann cell migration characteristics on PPy surface. Despite the wide application in the biomedical field, PPy is unsuitable for application alone because it is brittle, rigid, and nonbiodegradable. Therefore, many polymers have been tested in the fabrication of PPy/polymer composite material. Recently, an in vivo study was carried out to confirm the viability of PPy/polymer composite material as a scaffold for promoting peripheral nerve regeneration. Signs of PPy degradation were observed after 3 months after implantation, while a more significant reduction was seen after 6 months.^17^ However, to our knowledge, there is a scarcity of hitherto reports on the study of the biocompatibility of PPy/polymer composite nerve conduits in central nervous system (CNS) injuries.

Polypyrrole/polylactic acid (PPy/PLA) is a potential stem cell seeding biomaterial used for nerve tissue engineering.^18^, ^19^ Bone marrow stromal cells (BMSCs) are regarded as an ideal candidate type of cell for transplantation due to low immunorejection, rapid propagation, and easy accessibility.^20^, ^21^ Furthermore, BMSCs can release a series of factors that may provide trophic support and can differentiate into different types of cell, such as neurons, oligodendrocytes, and astrocytes, which can replace the lost tissue.^22^, ^23^ It has been shown that BMSCs are very beneficial in SCI.^24^ In this study, the efficacy of a PPy/PLA nanofibrous scaffold was examined for spinal cord injury treatment, and BMSCs were applied to optimize the functional recovery.

## 2 MATERIAL AND METHODS

### 2.1 PPy/PLA nanofibrous scaffold preparation

Shandong Institute of Medical Instruments, China, supplied PLA with an average molecular weight (Mw) of 130 000 g/mol (inherent viscosity 0.97 dL/g). Pyrrole (98%) was obtained from Sinopharm Chemical Reagent Co. Ltd., China. P123 (EO20PO70EO20, Mw = 5800) was purchased from Sigma‐Aldrich, USA. Ferric chloride (FeCl3), dichloromethane (DCM), N,N‐dimethylformamide (DMF), ethanol, and other solvents were supplied by Beijing Chemical Works, China. All the materials were used without further purification.

Pyrrole was polymerized with P123 according to our previous work. Briefly, 2.3 g of P123 was completely dissolved in 230 mL of deionized water to form the P123 solution. The solution was then mixed with 1.59 mL of pyrrole monomer using vigorous stirring for 1 hour. Consequently, an aqueous FeCl3 solution (1 g/mL) was added to the mixture while stirring to trigger an oxidative polymerization. The polymerization continued for 6 hours at 18°C. PPy nanoparticles were collected through centrifuge and were washed with deionized water and alcohol several times before they were vacuum‐dried at 60°C for 24 hours and ground to fine powders for further use.

Polypyrrole nanoparticles were homogeneously dispersed in DMF by ultrasonication at a concentration of 5.6% (w/v) and then were stirred continuously overnight at room temperature to form the PPy suspension. PLA was dissolved in DCM at a concentration of 18.75% (w/v). The PPy suspension or pure DMF was mixed with the PLA solution while stirring to the point where DCM:DMF reached 2:1, in order to achieve the PPy/PLA suspension or the PLA solution, respectively.

The electro‐spinning device (Ucalery Co., Ltd.) was set up with a homemade high‐speed collecting drum. The prepared PPy/PLA suspension or PLA solution was added into a plastic syringe with a stainless‐steel needle. The outer diameter of the needle was 0.6 mm. The flow rate of the suspension was 1 mL/h. The applied voltage was 15 kV. The aligned films were achieved at the speed of 2500 r/min. The electrospun films were dried in a vacuum drier at 40°C for 3 days.

### 2.2 Animals and BMSCs

Female Sprague‐Dawley rats, 200‐250 g, were obtained from Vital River Laboratories, China. All the animals were housed in an environment with temperature of 22 ± 1°C, relative humidity of 50% ± 1%, and a light/dark cycle of 12/12 hours All animal studies (including the rats euthanasia procedure) were done in compliance with the regulations and guidelines of Beijing Neurosurgical Institute institutional animal care (Permit Number: 201301020). Efforts were made to minimize animal suffering during the procedures.

Rats were anesthetized through intraperitoneal injection of chloral hydrate solution (0.4 mL/100 g). BMSCs were collected from femurs and tibias out of adult Sprague‐Dawley rats. The cells were plated at a density of 1 × 10^5^ per cm^2^ in Dulbecco's modified Eagle's medium/nutrient mixture F‐12 (GIBCO, USA) supplemented with 15% fetal bovine serum (GIBCO, USA), in a humidified atmosphere containing 5%CO_2_/95% air at 37°C. The medium was replaced with a new one every third day. The cells were digested with trypsin (GIBCO, USA) and passaged upon reaching 90% confluence. After five passages, cells were suspended with normal saline to a concentration of 1 × 10^5^ cell/mL. The percentage of cell viability was determined by the average counts from five different randomly chosen microscopic fields. Flow cytometry was performed to detect the cell purity. If the percentage of CD105, CD90, and CD73 were all higher than 95%, and the percentage of CD45 and CD34 were lower than 5%, the cells were used for transplantation.

### 2.3 Establishment of rat model with complete transected spinal cord and the transplantation of BMSCs

**Figure 1:**
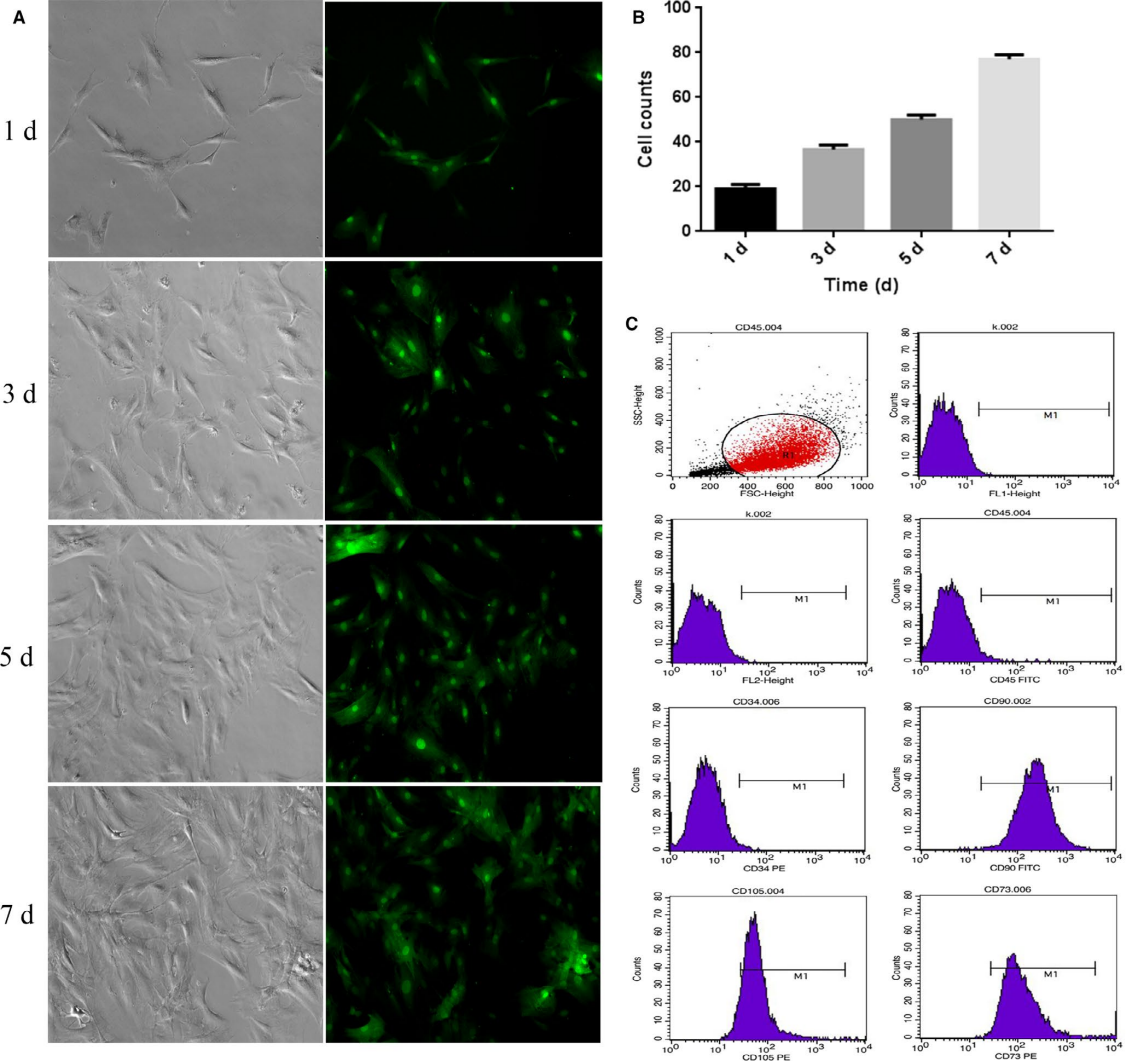
The in vitro culture of GFP‐BMSCs observed by fluorescence microscope and Immuno‐phenotypes of BMSCs. A, GFP‐BMSCs cultured in media from day 1 to day 7. B, Cell counts of GFP‐BMSCs cultured from day 1 to day 7. C, CD45 negative (1.62%), CD34 negative (0.69%), CD105 positive (90.74.%), CD90 positive (99.91.%), CD73 positive (98.49.%). Scale bar = 50 μm

The rats were randomly divided into three groups (n = 18/group: control group, PPy/PLA group, and PPy/PLA/BMSCs group). Briefly, all animals were subjected to intraperitoneal anesthesia using 10% chloral hydrate solution (0.4 mL/100 g). A 2.5‐cm midline skin incision was then performed along the vertebrae T7‐T10. The thoracolumbar fascia and paraspinal musculature were incised along the spinous processes and retracted. A laminectomy was performed at T7‐T9 vertebrae level under a surgical microscope. A ~2.5 mm‐long block of spinal cord at the center of T7‐9 vertebrae level (T8 spinal segment) was cut and removed using iridectomy scissors, with visual verification to ensure complete transection ventrally and laterally. The material implant was sized to adapt the dimension of the cavity (Figure 1B‐C). Rats in PPy/PLA/BMSCs group were transplanted with 1 × 10^5^ BMSCs after implantation of PPy/PLA, while those in the PPy/PLA group were implanted with PPy/PLA only; no implantation was performed in a control group. Then, the incision was closed by suturing the muscles and the skin in order. Antibiotic (penicillin 10 000 units) was given after the surgery, and the rats were observed until they were all conscious. Manual bladder and peritoneal exercise were performed twice a day until the recovery of the bladder and intestinal reflex.

### 2.4 Neurobehavioral testing and electrophysiology

The locomotor recovery of the animals was assessed by two independent observers in an open field (diameter of plastic pool: 90 cm) using the 21‐point Basso, Beattie, and Bresnahan (BBB) open‐field locomotor score from 1 to 6 weeks after SCI. The BBB scale was used to assess hind limb locomotor recovery including joint movements, stepping ability, coordination, and trunk stability. The function of both hind limbs was detected. Rats were tested once a week using a blind approach, and the duration of each session was 2 minutes per rat.

Motor evoked potential (MEP) was measured using the Neurosoft electrophysiology monitoring system (neuron spectrum 5, Russia). MEP was obtained using stainless‐steel needle electrode, Neurosoft software. Briefly, rats were anesthetized with 10% chloral hydrate solution (0.4 mL/100 g). The stimulating electrode was then inserted into the scalp, and the recording electrode was introduced into the tibial muscle. The reference electrodes were inserted subcutaneously above the nostril, the peritoneum, and the tail. MEP was obtained by electrical stimulation at 0.1 ms duration, 10 mA intensity, and at a frequency of 1 Hz, and the latent period of MEP was analyzed.

### 2.5 Tissue processing for light and fluorescent microscope

After 6 weeks, the rats were anesthetized using intraperitoneal injection of 10% chloral hydrate solution (0.4 mL/100 g). The T6‐T10 spinal cords were then collected (six specimens/group), fixed in 4% paraformaldehyde containing 30% sucrose at 4°C for 48 hours, and embedded in OCT (Sakura Finetechnical, Japan). Samples were then divided into 20‐µm sections, which were stained with hematoxylin and eosin for histological examination. Immunofluorescent staining was performed to assess nerve regeneration, spared neurons, survival, and differentiation of transplanted BMSCs.

### 2.6 Histological analysis

For histological analysis, the spinal cord specimens were managed the same as above reported. Immunofluorescent staining was performed on 20‐μm sections, which were stained with the following antibodies: green fluorescence protein (GFP, 1:200, A10262, Molecular probes); neurofilament (NF, 1:200, Ab8135, Abcam); caspase 3 (Casp3, 1:200, Abcam); neuron‐specific marker (NeuN, 1:200, Ab104224, Abcam), ß‐tubulin III (Tubulin; 1:100, T8578, Sigma); glial fibrillary acidic protein (GFAP, 1:400, Ab10062, Abcam); oligodendrocyte‐specific antibody (O4, 1:200, Ab7474, Abcam); and 4′,6‐diamidino‐2‐phenylindole (DAPI, 1:1000, D1306, Invitrogen). Sections of spinal cord were imaged at 10×, 20×, and 40× magnifications using a confocal microscope (Leica SP5, Germany). GFP staining was performed to check the survival of the BMSCs. NF staining was used to detect axon growth in the injured area. NeuN staining was performed to detect the spared neurons in the area adjacent to the lesion. Double immunofluorescent assay (GFP/Tubulin, GFP/GFAP, GFP/O4) was performed to determine whether the GFP^+^ cells (include the BMSCs) differentiate into other types of cell.

### 2.7 Ultra‐structure analysis by both transmission and scanning electron microscope

Six weeks postimplantation, the spinal cords (six specimens each group) were examined by transmission electron microscopy (Hitachi TEM, Japan). The rats were anesthetized as mentioned above. Then, the heart was exposed. Next, 300 mL of 1× PBS followed by 300 mL 4% paraformaldehyde was perfused through the vascular system. The spinal cord tissue was fixed with 2.5% glutaraldehyde and 2% paraformaldehyde. Ultra‐thin sections were achieved with an ultramicrotome (Leica EM UC6, Germany) and stained with uranyl acetate and lead citrate. Observation was performed by TEM (Hitachi). The PPy/PLA nanofibrous scaffold was examined by scanning electron microscopy (Hitachi TM‐100 SEM, Japan).

### 2.8 Quantitative real‐time RT‐PCR

Total RNA was extracted from the spinal cord tissue (six specimens each group) including the graft sites using the Trizol reagent (Invitrogen). RNA concentrations were determined by the absorbance readings at 260 nm with Gene Quant (Amersham Biosciences, Amersham, UK), and 1 μg of total RNA was reverse transcribed to cDNA using the Revert Aid TM First Strand cDNA Synthesis Kit with oligo‐dT primers (Fermentas, Hanover, MD, USA). PCR amplification was performed in a 50 μL volume containing 2 μL of each primer and 25 μL of SYBR Green qPCR mix (Toyobo, Osaka, Japan). The amplification procedure consisted of three processes: 50°C for 2 minutes; 95°C for 10 minutes and 40 cycles of amplification reactions at 95°C for 15 seconds; and 60°C for 1 minutes. The primer sequences of genes used in PCR are shown in Table 1. Glyceraldehyde‐3‐phosphate dehydrogenase (GAPDH) cDNA was used as a control. The relative expression between a given sample and a reference sample was calculated using the 2^−ΔΔCt^ method.

**Table 1:** Primer sequences used in qRT‐PCR

Gene name	Forward primer	Reverse primer
Casp3	5′‐ACTGGAAAGCCGAAACTCTTCATCA‐3′	5′‐GGAAGTCGGCCTCCACTGGTATC‐3′
Cspg4	5′‐GCCTCCACTCAGCCATTCTCA‐3′	5′‐GCCAACTTCATCACCAGCAGAG‐3′
MMP2	5′‐CCAAGAACTTCCGACTATCCAATGA‐3′	5′‐CAGTGTAGGCGTGGGTCCAGTA‐3′
Nefh	5′‐CCTGGATATTGAGATCGCTGCTTAC‐3′	5′‐TGTCACTTCTTCTGTCACCTGGAT‐3′
VEGFA	5′‐CCAGGCTGCACCCACGACAG‐3′	5′‐TCATTGCAGCAGCCCGCAC‐3′
GAPDH	5′‐TGGAGTCTACTGGCGTCTT‐3′,	5′‐TGTCATATTTCTCGTGGTTCA‐3′

### 2.9 Quantitative analysis

Immunofluorescence imaging was used to analyze the morphological aspects of each experimental group. All quantitative cell analyses were performed in a blinded fashion. For quantitative analyses of the glial scar, new regenerated axons, neuron apoptosis, and survival of transplanted cells, GFAP^+^, NF^+^, Casp3^+^, NeuN^+^, and GFP^+^ were counted in longitudinal sections (section thickness = 20 µm). The quantification of positive immunostaining was achieved from six specimens, each with five sections and four different randomly chosen microscopic fields (region of interests, 0.775 × 0.775 mm^2^) at the lesion site. The sections were examined under a confocal laser scanning microscope at 200× and 400× magnification (Leica SP5, Germany) 6 weeks postoperation. Cells and new generated axons were counted with ImageJ Software. The length of the lesion was calculated from each of five different sections with six specimens H&E stained sections under a light microscope at 10× magnification. The data of the new generated axons in TEM results were obtained from six specimens, each with five sections and four different randomly chosen fields, under 7000× magnification.

### 2.10 Statistical analysis

All the data were expressed as the mean ± standard deviation (SD). Statistics were calculated with the software SPSS 17.0. Differences between groups were compared with one‐way ANOVA, Fisher's least significant difference (LSD) method was used for the post hoc test. The differences in the repeated measurements were analyzed with multivariate ANOVA. A significant difference was indicated by *P* < 0.01 and *P* < 0.05.

## 3 RESULTS

### 3.1 In vitro culture and characterization of BMSCs

Bone marrow stromal cell were collected from femurs and tibias of adult Sprague‐Dawley rats. Cells were collected after adherence and were then cultured (Figure 1A) and passaged for five times. The cell purity was detected by flow cytometry (FCM). In vitro culture showed a rapid proliferation of BMSCs (Figure 1B). The phenotypes of BMSCs used in our study were positive for CD105 (90.74%), CD90 (99.91%), and CD73 (98.49%) and negative for CD45 (1.62%) and CD34 (0.69%); Figure 1C).

### 3.2 Structure of the PPy/PLA nanofibrous scaffold and implantation of the scaffold

**Figure 2:**
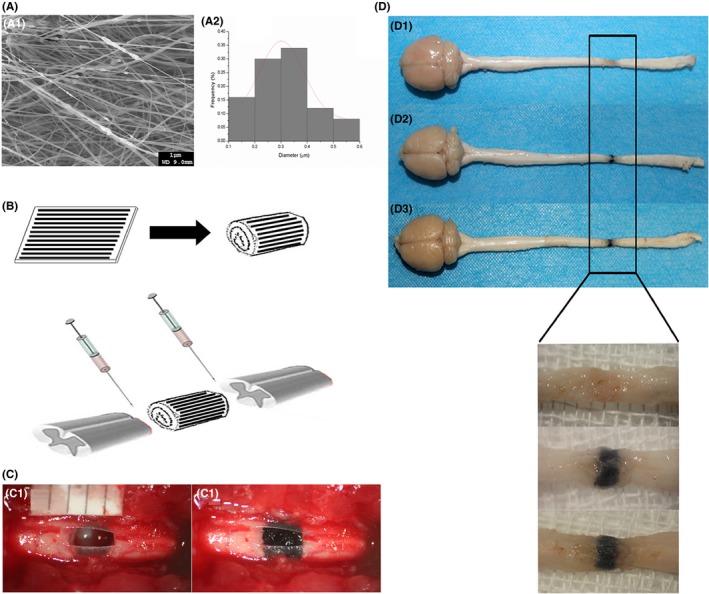
(A1) SEM image of the aligned PPy/PLA nanofibrous scaffold with distribution of PPy particles in the fibers. Scale bar = 1 μm; (A2) histogram of the diameter distribution of the nanofibrous scaffold. B, The aligned (structure illustrated with black stripe) PPy/PLA nanofibrous scaffold was rolled, then the PPy/PLA was implanted into the transection site and the BMSCs were transplanted between the normal tissue and the PPy/PLA nanofibrous scaffold; (C1) Complete transection of the spinal cord; (C2) implantation of the PPy/PLA nanofibrous scaffold. D, Dorsal view of the representative spinal cords in different group, (D1) control without any treatment, (D2) PPy/PLA receiving group, (D3) PPy/PLA/BMSCs group

We first tested the morphology of the electrospun nanofibers containing PPy nanoparticles. As indicated by the scanning electron microscope (SEM), the nanoparticles were dispersed within the fiber networks (Figure 2a1). PPy nanoparticles were located inside the nanofibers causing roughness on the fiber surfaces. From the quantitative analysis of the fiber diameter distribution (Figure 2a2), the average fiber diameter (D avg) was 301.79 ± 85.45 nm, which is larger than the average diameter of the PPy nanoparticles (85.9 ± 0.8 nm), and which suggests that it can insure the embedding of the PPy nanoparticles. A ~2.5 mm‐long block of spinal cord was surgically removed to create a complete transected lesion. The PPy/PLA nanofibrous scaffold was sized to adapt to the lesion cavity (Figure 2B‐C). After 6 weeks of implantation, the PPy/PLA nanofibrous scaffold was integrated well with the host spinal cord tissue (Figure 2d1‐d3).

### 3.3 The recovery of electrophysiology and the assessment of functional recovery in SCI

**Figure 3:**
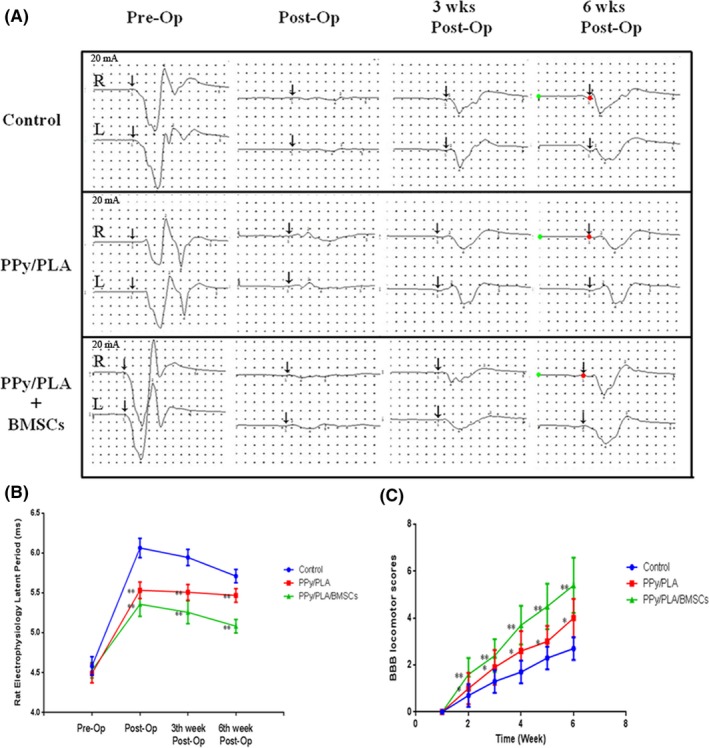
A, B, The electrophysiological recovery postoperation. The latent period of the PPy/PLA/BMSCs group showed significant improvement compared with the PPy/PLA and control groups (the length of the latent period was measured from green dot to red dot). C, The behavioral assessment using the BBB locomotor rating scale. The BBB analysis was performed continually starting from the first week after operation. BBB analysis indicated that the PPy/PLA/BMSCs‐treated group showed the greatest functional recovery compared with the control group or those treated with PPy/PLA alone. **P < 0.01, *P < 0.05

Motor evoked potentials (MEPs) assay was performed before surgery, and at 3 and 6 weeks after operation to assess the functional recovery of the injured spinal cord. The short latent period in the MEP assay is considered a normal nerve conduction. Immediately after injury, the rats treated with PPy/PLA/BMSCs (5.36 ± 0.15) and PPy/PLA (5.53 ± 0.10) showed a shorter latent period of the lower extremity compared with the control group (6.06 ± 0.12, ^**^ *P* < 0.01). At the 3‐week postoperation, a slight yet significant improvement was observed in each group. In addition, 6 weeks after the operation, the PPy/PLA/BMSCs group showed a shorter latent period (5.08 ± 0.09) compared with PPy/PLA (5.47 ± 0.09) and compared with control groups (5.71 ± 0.08) (Figure 3A,B). These data verified the role of PPy/PLA and BMSCs in the electrophysiological improvement of SCI.

Functional recovery is an important indicator for the SCI recovery. The BBB locomotor rating scores were applied to evaluate the motor functional recovery in rats after treatment. Immediately after operation, rats showed no motor function in both lower limbs, indicating the loss of motor function after injury. The motor function in the PPy/PLA and PPy/PLA/BMSCs groups recovered gradually with increased BBB scores at different observation times compared the control group. After 6 weeks postinjury, the control rats showed limited self‐healing and had slight improvement of motor recovery with corresponding mean average BBB scores (2.7 ± 0.48). Meanwhile, the PPy/PLA treatment group showed persistent locomotion recovery and achieved mean average BBB scores (4 ± 0.82). Moreover, the PPy/PLA/BMSCs group showed the best performance in locomotion among these three experimental groups with the mean average BBB scores (5.4 ± 1.17) (Figure 3C). Again, the implantation of the PPy/PLA nanofibrous scaffold with BMSCs could markedly improve locomotor functional recovery.

### 3.4 PPy/PLA nanofibrous scaffold combined with BMSC transplantation reduces the scar tissue formation

**Figure 4:**
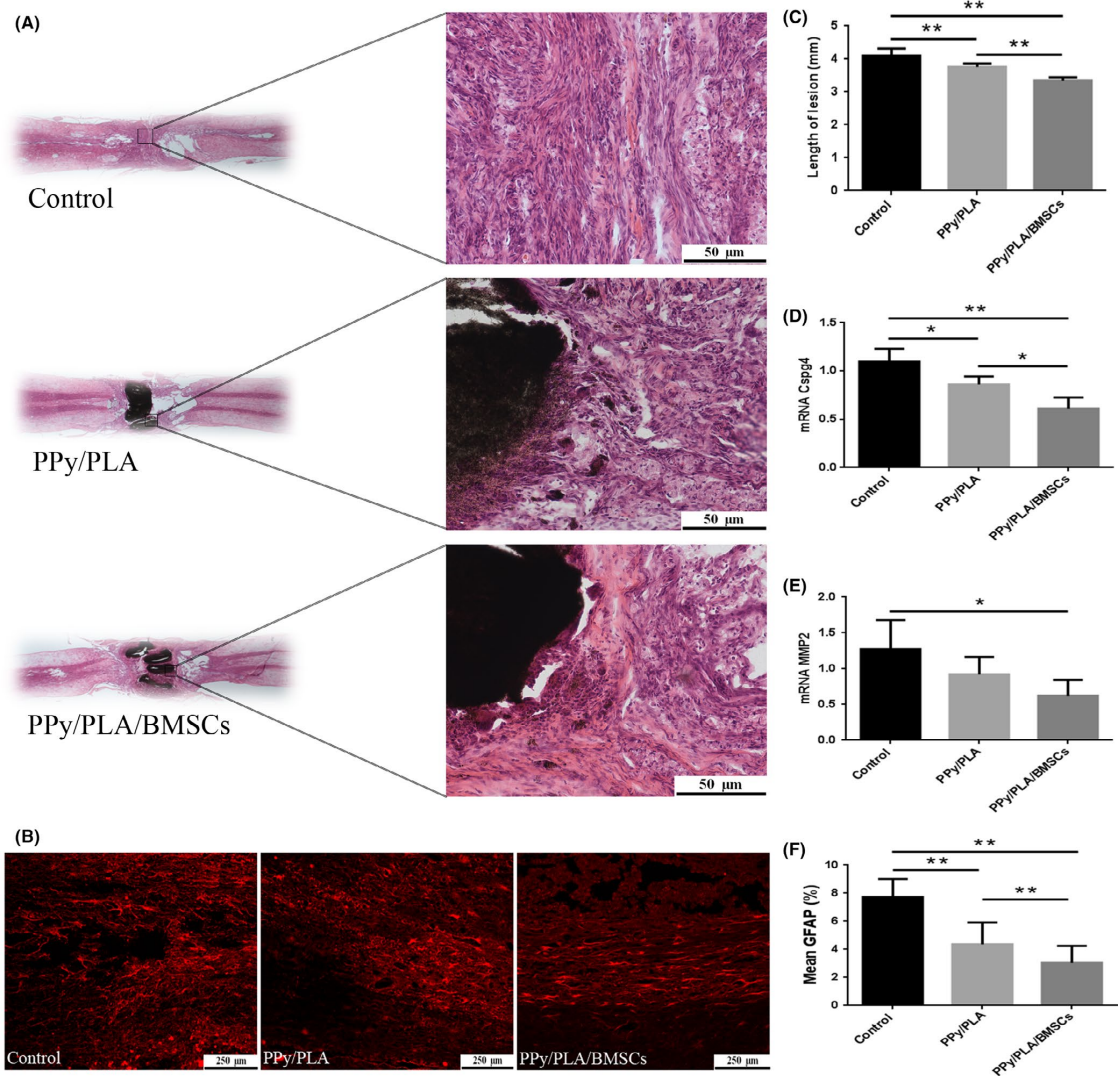
A, Hematoxylin‐eosin staining histology tissue images of each group. B, Immunostaining with GFAP antibody in each group. C, Quantified length of the lesion area; the lesion volume in the PPY/PLA and PPY/PLA/BMSCs receiving group was significantly decreased compared with control group. D, The mRNA expression of Cspg4 (Chondroitin sulfate proteoglycan 4) showed significant difference in the PPY/PLA and PPY/PLA/BMSCs groups. E, The mRNA expression of MMP2 (Matrix metallopeptidase 2) showed a significant difference in the PPY/PLA/BMSCs receiving group. F, Quantitative analysis of GFAP. **P < 0.01 vs the control group, *P < 0.05 vs the control group; scale bar in (A) = 1000 μm, scale bar in magnification = 50 μm

Six weeks postoperation, hematoxylin and eosin (HE) analysis was performed on longitudinal sections of the injured spinal cords. Scar tissue filled the lesion defect in the control group, while in the other two groups, the PPy/PLA nanofibrous scaffold filled the lesion and integrated well with the host tissue (Figure 4A). Decreased accumulations of GFAP‐positive adjacent to the injured area were found in the PPy/PLA (4.36 ± 1.56) and PPy/PLA/BMSCs group (3.04 ± 1.19) compared with control group (7.73 ± 1.30) (Figure 4B,F). The scar length was obviously reduced in the treatment group (Figure 4C). In addition, the qPCR result showed that the transcription of Cspg4 mRNA in the PPy/PLA/BMSCs‐treated group (0.61 ± 0.12) was significantly lower than PPy/PLA (0.86 ± 0.08) and control groups (1.10 ± 0.13) (Figure 4D); while, MMP2 mRNA in the PPy/PLA/BMSCs‐treated group (0.62 ± 0.22) was significantly reduced compared with PPy/PLA (0.92 ± 0.24) and control groups (1.27 ± 0.41) (Figure 4E). The above data suggested that PPy/PLA nanofibrous scaffold implantation and BMSCs transplantation can reduce the scar formation following SCI.

### 3.5 Axon regeneration and remyelination in the injured area

**Figure 5:**
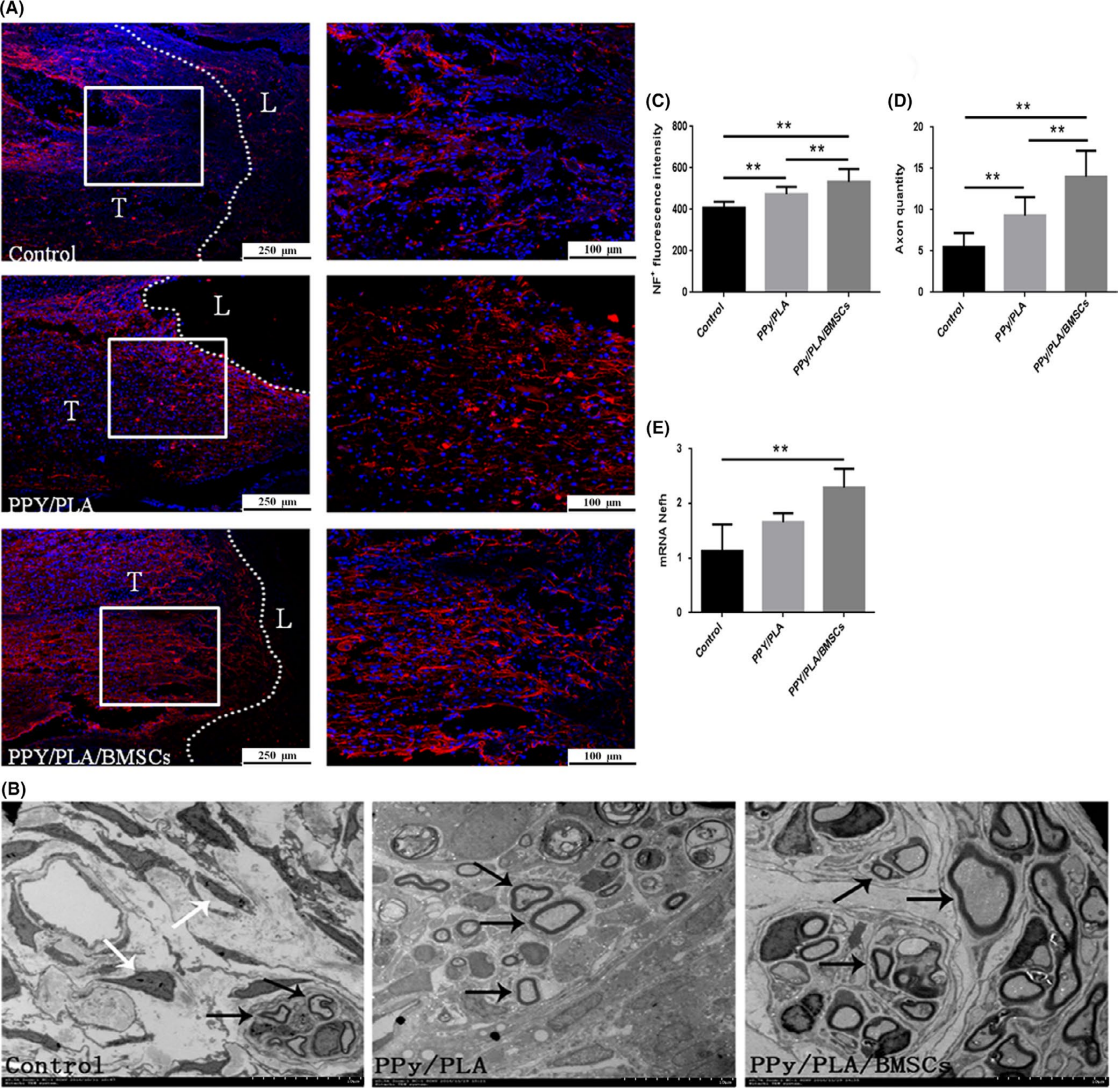
A, The immunofluorescence assay of nerve fibers stained with NF (red) in longitudinal sections at 6 wk after SCI. The dotted line marks the border between the host spinal cord and the lesion area (T, host tissue; L, Lesion area). Scale bar = 250 μm, scale bar in all magnification = 100 μm. B, The detection of newly generated myelinated axons by TEM 6 wk after transplantation. Few myelinated axons were detected in the control group (indicated by black arrow), and many fibers were detected in the control group (indicated by white arrow). Many newly generated myelinated axons were detected (indicated by black arrow) in the PPy/PLA and PPy/PLA/BMSCs groups, scale bar = 10 μm. The quantification of NF^+^ fluorescence intensity in the three groups is shown in (C). The quantification of myelinated axon between the three groups is shown in (D). Quantification of the mRNA expression of Nefh (neurofilament, heavy polypeptide) is shown in (E). **P < 0.01, *P < 0.05

Axon regeneration is essential for the nerve functional recovery. The effect of treatment on axonal sprouting following SCI was evaluated by neurofilament (NF) staining. Axon regeneration at the lesion site of each experimental group was quantified by analyzing the fluorescence intensity of NF staining, as defined by the injured border. Rats treated with PPy/PLA/BMSCs showed higher NF‐positive signals (533.57 ± 62.16; ^**^ *P* < 0.01) compared with the control group (407.20 ± 29.71) and the PPy/PLA‐administered group (474.42 ± 34.40) (Figure 5A,C). Furthermore, qPCR was used to confirm the new axon formation. Six weeks after implantation, the Nefh mRNA in the PPy/PLA/BMSCs‐treated group (2.39 ± 0.40; ^**^ *P* < 0.01) was significantly higher compared with the control (1.28 ± 0.47) and PPy/PLA groups (1.66 ± 0.16) **(**Figure 5E). Based on these data, we believe that treatment with PPy/PLA/BMSCs has the ability to enhance axonal regeneration within the lesion site, following SCI.

Myelin formation is another important process for the nerve function recovery. It has been reported that improving myelination is beneficial for functional recovery in spinal cord injury. Therefore, in order to investigate the efficacy of nanofibrous scaffold to promote myelin formation, TEM was employed and at week 6th post‐SCI. Briefly, rats treated with PPy/PLA/BMSCs had more myelinated axons (22.4 ± 1.1; ^**^P < 0.01) than those treated with PPy/PLA (14.6 ± 1.1) or the control group (8.6 ± 1.1) (Figure 5B,D).

### 3.6 PPy/PLA/BMSCs reduce neuron apoptosis and vascular formation in rat SCI

**Figure 6:**
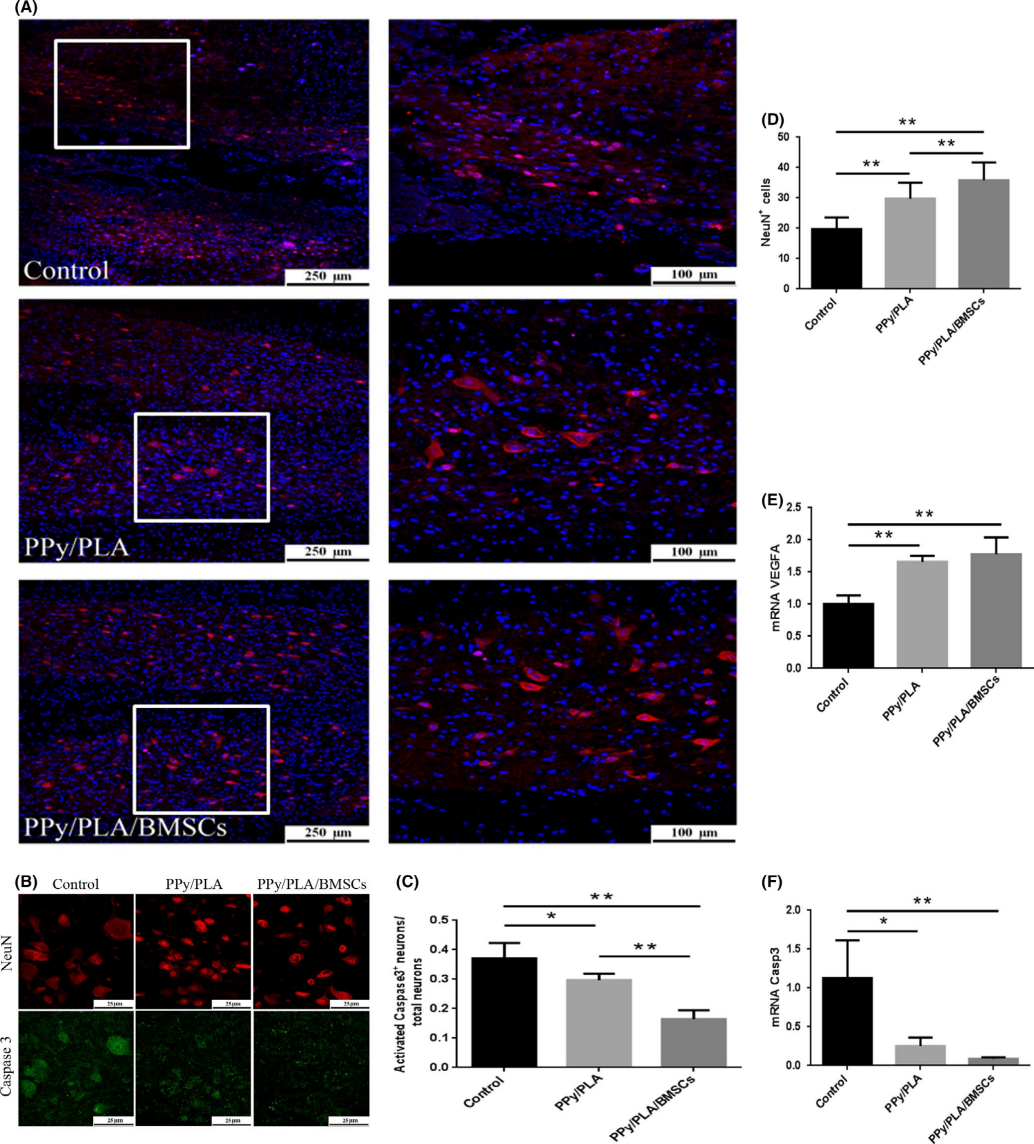
A, The immunofluorescence assay of neurons in the area adjacent to the lesion area stained with NeuN (red) in longitudinal sections at 6 wk after SCI. The image (B) and quantification (C) of caspase3‐positive staining within each group. D, Statistically significant differences in NeuN^+^ cells between the three groups. E, F, The mRNA expression of VEGFA (vascular endothelial growth factor A) and Casp 3 (caspase 3) showed a significant difference between three groups. **P < 0.01, *P < 0.05. Scale bar in (A) = 250 μm, scale bar in all magnification = 100 μm. Scale bar in (B) = 25 μm

The long‐term neurological deficits after spinal cord injury may be due in part to the widespread apoptosis of neurons in regions distant from and relatively unaffected by the initial injury. The immunofluorescence assay was performed to investigate the spared neurons adjacent to the lesion site of each experimental group by quantifying the NeuN‐positive cells. Six weeks after transplantation, rats treated with PPy/PLA/BMSCs showed higher NeuN‐positive cells (35.87 ± 5.81; ^**^P < 0.01). Both groups, that is, the control group (19.72 ± 3.906) and the PPy/PLA receiving group (29.78 ± 5.23) had lower number of NeuN‐positive cells compared with the PPy/PLA/BMSCs receiving group (Figure 6A,D). The mean fluorescence intensity per cell decreased significantly in the PPy/PLA (0.30 ± 0.02) and PPy/PLA/BMSCs group (0.16 ± 0.03) compared with the control group (0.37 ± 0.05) (^**^P < 0.01, ^*^P < 0.05; Figure 6B,C). In addition, the qPCR showed that the transcription of Casp3 mRNA in the PPy/PLA/BMSCs‐treated group (0.90 ± 0.02; ^**^P < 0.01) was significantly lower compared with the control group (1.13 ± 0.48) and the PPy/PLA group (0.25 ± 0.11) (Figure 6F). These data indicated that PPy/PLA and PPy/PLA/BMSCs might significantly reduce neuronal apoptosis following SCI.

Vascular endothelial growth factor (VEGF) is essential for endothelial cell functions associated with angiogenesis. Briefly, a significantly higher expression of VEGFA mRNA was found in the PPy/PLA/BMSCs‐treated group (1.78 ± 0.26; Figure 6E; ^**^P < 0.01) compared with the control (1.01 ± 0.13) and PPy/PLA groups (1.66 ± 0.10), which suggested that PPy/PLA/BMSCs can improve the microenvironment in the injured site by inducing new vascular formation.

### 3.7 In vivo survival and differentiation of transplanted BMSCs in SCI

**Figure 7:**
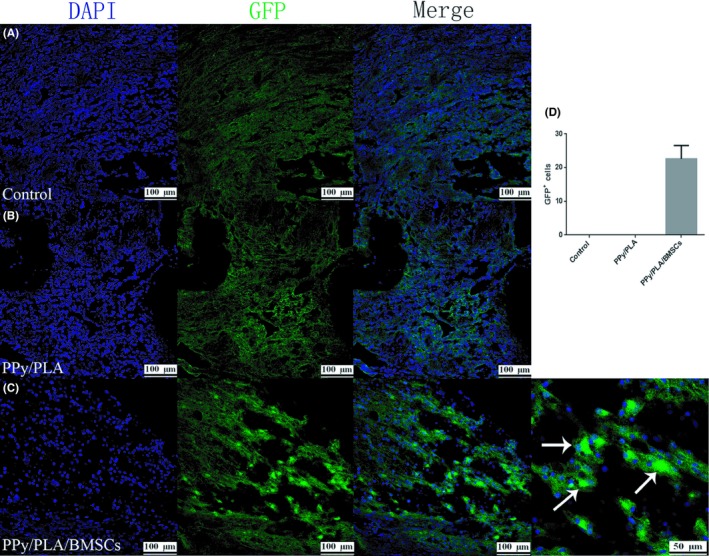
The in vivo survival of BMSCs. A, B, No obvious signal was detected in this control and PPy/PLA group. C, The signal of GFP‐BMSCs (indicated by arrow) was detected in the injured area that was the target site of transplantation. D, Quantification of GFP‐BMSC‐positive cells. Scale bar = 100 µm, scale bar in magnification = 50 µm

**Figure 8:**
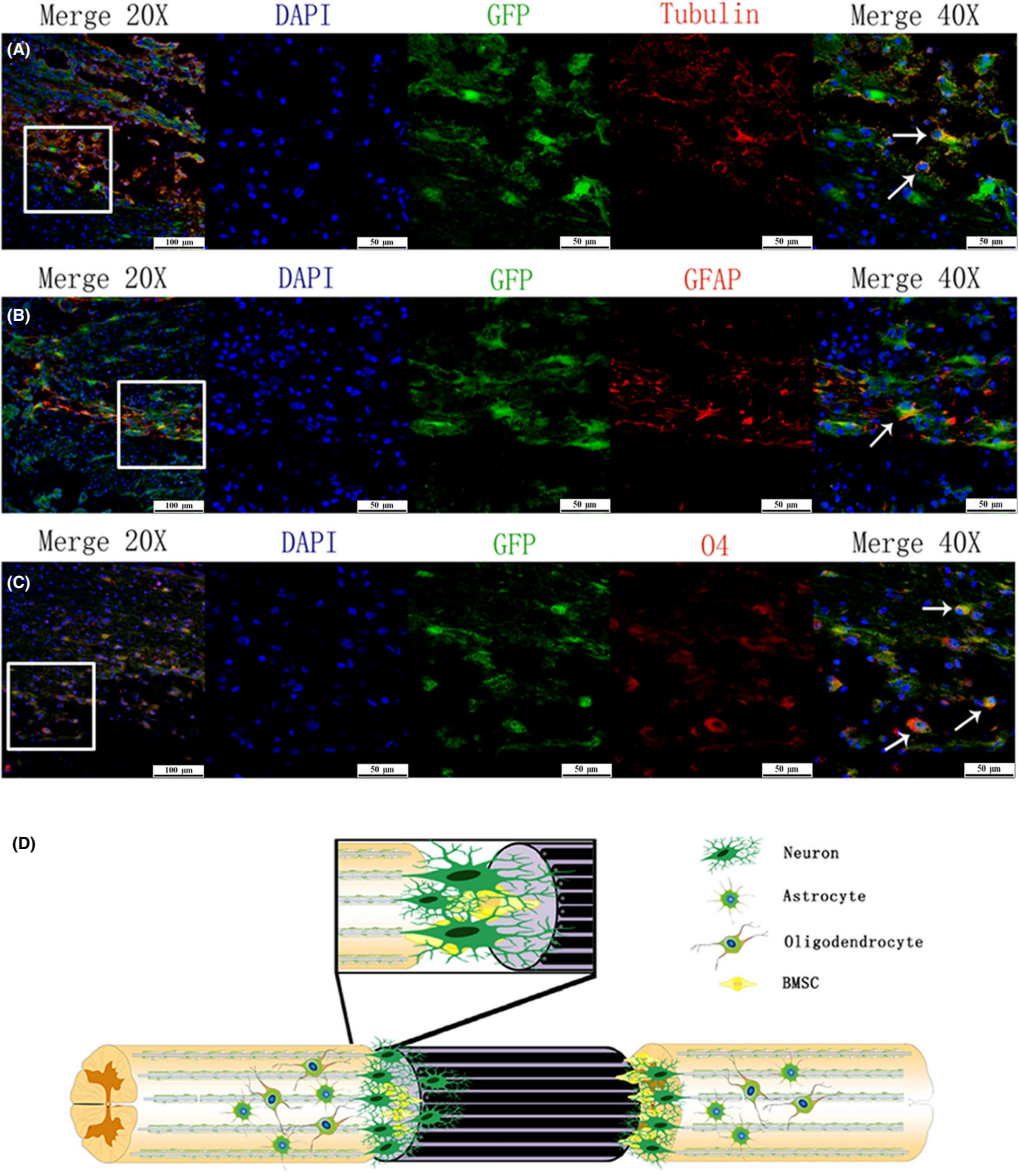
The in vivo differentiation of BMSCs into neural and neuroglial cells at 6 wk posttransplantation. Immunofluorescence micrograph showing the transplanted BMSCs (green fluorescent cells) are aggregated in the site of injury, immunofluorescence staining showing a few transplanted cells express markers for neural and neuroglial cells (indicated by arrow) (A) Tubulin (+): red, Nucleus: blue; (B) GFAP (+): red, Nucleus: blue; (C) O4 (+): red, Nucleus: blue. Scale bar in (A, B, C) = 100 µm, scale bar in magnification = 50 µm. D, Figure illustration of our treatment strategy using PPy/PLA/BMSCs to treat SCI. The PPy/PLA nanofibrous scaffold bridging both end lesions. The transplanted BMSCs not only supported the regeneration but also connected the host tissue and the implanted nanofibrous scaffold

Six weeks after the operation, immunofluorescence assay was performed to evaluate the survival of BMSCs labeled green fluorescent protein (GFP). No GFP^+^ cells were detected in the control and PPy/PLA group, while a significant GFP signal was found in rats transplanted with PPy/PLA/BMSCs (Figure 7). To further determine the fate of transplanted cells and whether they differentiate into different types of cell, we performed the immunofluorescence assay by using different cell markers. The transplanted cells presented several neuronal‐like features with the immunoexpression of Tubulin, an early stage neuron marker, while only a small number of cells expressed GFAP (a marker of astrocytes) and O4 (a marker of oligodendrocytes), which confirms that the GFP^+^ cells survived and differentiated into neural and neuroglial cells (Figure 8A‐C). The differentiation of transplanted BMSCs into neural and neuroglial cells may also provide an important role to support functional recovery in SCI rats.

## 4 DISCUSSION

Given that spontaneous axonal regeneration after spinal cord injury is limited,^25^ considerable efforts have been made to break this limitation. The efficacy of the PPy/biodegradable polymer in promoting nerve regeneration has been reported in previous studies. The majority of these studies are about peripheral nerve injury treatment. Xu et al^17^ have used PPy/PDLLA to repair sciatic nerve injury in rats. PPy/PDLLA conduits were applied to bridge a 10 mm defect in the sciatic nerve of rat. After 6 months of observation, the rats with the PPy/PDLLA conduits showed functional recovery similar to the autologous nerve graft. In a different study performed by Zhang et al^26^ conductive scaffold material combined with growth factors was evaluated to achieve better functional recovery instead of simple implantation of the scaffold. Unlike previously reported studies, in the present study, we transplanted rat BMSCs immediately after implantation of the PPy/PLA nanofibrous scaffold and the results indicated that this combination therapy could improve neurological function recovery in the injured spinal cord of rats.

After injury, a sequence of progressive pathological changes occurs. The lesion cavity is gradually filled by scar tissue, which becomes a permanent barrier for nerve regeneration.^27^, ^28^ In our study, implantation of PPy/PLA nanofibrous scaffold resulted in downregulation of the chondroitin sulfate proteoglycan, which is a strong axon regeneration inhibitor molecule in the extracellular matrix in the injured area.^29^ Moreover, transplantation of BMSCs has shown to suppress glial scar formation in rat spinal cord injury.^30^ In the present study, BMSCs transplantation caused the significant downregulation of this inhibitory molecule. Our data result suggested that this combined strategy can reduce glial scar formation after spinal cord injury, allowing the new axons to pass through the lesion,^31^ and thus, recovering the nerve conduction. In addition, these results were confirmed by the electrophysiology assessment, which revealed a shorter latent period in the PPy/PLA/BMSCs group compared with the other two groups.

In the current study, the PPy/PLA/BMSCs group showed the best performance according to BBB scores. We attributed these surprising results to several factors that induce the recovery of the locomotor function. First, the PPy/PLA nanofibrous scaffold fills the lesion defect and reduces scar formation, which benefit axonal regeneration and remyelination. Although PLA itself can promote nerve regeneration,^32^ the nerve conduction velocities (NCVs) of regenerated nerves are less than that in healthy nerves.^17^ Therefore, a conductive material such as PPy is important to recover the electrophysiological properties of the nerve and to achieve NCV that is within the healthy nerve range, which is necessary to reach better functional recovery. Xu et al have reported that PPy/PDLLA group showed greater NCV than PDLLA group (66.59 ± 7.97 m/s vs 51.54 ± 0.66). Second, PLA is a polycationic conducting polymer, which may attract cells to the lesion area. Mihardja et al^33^ have suggested that the presence of PPy may restore the conduction of electrical impulses in myocardial infarction, thereby facilitating the growth and differentiation of the cells responsible for vascular formation. Third, electrical conduction conveyed by the scaffold may activate the voltage‐gated ion channels on cell membranes and induce a modulation in the intracellular signaling pathways, which in turn may impact the cell behavior, such as cell apoptosis.^34^ Last but not the least, angiogenic factors, such as VEGF, also have neuroprotective effects.^35^ Increased expression of VEGF and its receptor during hypoxic/ischemic injury to the brain and spinal cord suggests that VEGF could have a neuroprotective role in these pathophysiological processes.^36^ A study by Rong et al^37^ has shown that improving the expression of VEGF may reduce the expression of caspase‐3 protein, thus promoting nerve repair following SCI. Our results are consistent with the previous study, which suggest that this treatment strategy may provide neuroprotection.

In the present study, BMSCs transplantation had an important role in optimizing functional recovery. Lu et al^38^ have shown that the axons from transplanted graft cells extend in large numbers and over remarkably long distances to form connections with the host axons. Herein, we found that the transplanted BMSCs survived and were differentiated into neural and neuroglial cells at 6 weeks after transplantation. These differentiated cells may form a new network connection and restore the local neuronal connectivity^39^ by connecting the host tissue and the implanted nanofibrous scaffold as described in Figure 6D. Nonetheless, further investigation is needed to verify the interaction between host tissues, transplanted cells, and the scaffold. However, the significant improvement in functional recovery in the PPy/PLA/BMSCs group supports this hypothesis.

## 5 CONCLUSIONS

An aligned PPy/PLA nanofibrous scaffold was fabricated and grafted into a complete transected spinal cord to promote nerve regeneration and to recover nerve conduction. The results showed that the PPy/PLA nanofibrous scaffold can inhibit scar tissue formation and induce the axonal regeneration and myelination in the lesion area. New vascular formation was also promoted after implantation, creating a better microenvironment, which reduced neuronal apoptosis. Furthermore, the transplanted BMSCs survived and were differentiated into neural and neuroglial cells. This work demonstrated the remarkable potential of the implantation of the PPy/PLA nanofibrous scaffold combined with BMSCs transplantation for nerve regeneration and for motor functional recovery following SCI.

## CONFLICT OF INTEREST

The authors have no commercial, proprietary, or financial interest in the products or companies described in this article.
